# Pattern of activation of human antigen presenting cells by genotype GII.4 norovirus virus-like particles

**DOI:** 10.1186/1479-5876-11-127

**Published:** 2013-05-24

**Authors:** Eleonora Ponterio, Annacarmen Petrizzo, Ilaria Di Bartolo, Franco Maria Buonaguro, Luigi Buonaguro, Franco Maria Ruggeri

**Affiliations:** 1 Department of Veterinary Public Health and Food Safety, Istituto Superiore di Sanità, V.le Regina Elena, 299, 00161, Rome, Italy; 2 Laboratory of Molecular Biology and Viral Oncology, Department of Experimental Oncology, Istituto Nazionale per lo Studio e la Cura dei Tumori “Fondazione Pascale” - IRCCS, Via Mariano Semmola 142, Naples, 80131, Italy

**Keywords:** Norovirus, Immunology, PBMCs, VLPs

## Abstract

**Background:**

Virus-like particles (VLPs) from an Italian GII.4 norovirus strain were used to investigate activation and maturation of circulating antigen presenting cells (APCs) of human origin.

**Methods:**

Peripheral blood mononuclear cells (PBMCs) isolated from five healthy subjects were pulsed *ex vivo* with VLPs, and stained with a set of monoclonal antibodies (MAbs) for phenotypic analysis by flow cytometry. Cytokine release in cell supernatants was investigated by ELISA.

**Results:**

Norovirus VLPs induced activation and maturation of circulating APCs derived from the five donors, as well as production of IL-6, IFN-γ and TNF-α cytokines.

**Conclusions:**

The present results suggest that VLPs can activate antigen presenting cells for an efficient induction of the adaptive immune response.

## Background

Norovirus is the most important agent of gastroenteritis outbreaks worldwide, representing a relevant problem of public health [1,2]. In Europe, a recent report of the EFSA authority recognized the increasing role of norovirus in causing gastroenteritis outbreaks including foodborne [3].

Norovirus (NoV) has a single stranded RNA genome, organized in three ORFs, coding for non-structural proteins (ORF1), the major capsid protein (ORF2), and for a protein (ORF3) of still unknown function. Based on nucleotide sequences, NoVs have been classified into five genogroups (GI - GV), two of which (GI and GII) include at least 25 distinct genotypes and the majority of strains responsible for human infections [4]. Genotype GII.4 is the one most frequently associated with gastroenteritis outbreaks worldwide [5]. This genotype recurrently undergoes genetic changes, generating new variants [4].

Norovirus infection occurs at all ages, suggesting that a long-lasting protective immune response is not elicited, possibly related to a mostly strain-specific antibody response [4]. However, mechanisms of immunity toward norovirus are poorly understood, and data available on cross-protection following natural infection are contradictory [4,5].

Due to the absence of cell culture systems, most information on NoV has been obtained through the expression of the viral capsid protein in eukaryotic systems, particularly baculovirus. In insect cells, baculovirus recombinant capsid proteins self-assemble into virus-like particles (VLP) [4,5], thus providing suitable particulate antigen for diagnostic assays, for viral antigen characterization and for investigating norovirus-induced immune response [6,7]. X-ray crystallographic analysis of the recombinant capsid indicated that VLPs are composed of 180 copies of monomeric protein, which is structurally divided into a shell (S) and a protruding (P) domain, where most variable sequences are located. The latter interacts with both antibodies and cell receptors, including the histo-blood group antigens (HBGAs) [5,8-10]. High variability was described for HBGA affinity binding, depending on viral genotype [11]. Besides norovirus, the generation of VLPs has also been largely used to study the immune response towards other fastidious or complex viruses [12], and for production of vaccines against different human viruses, including HIV, papillomaviruses and rotavirus [13-15].

Although human norovirus strains cannot cross the species barrier and infect mice, injection with a low dose of human norovirus VLPs is effective at stimulating IgG and IgA responses in these animals [16]. Preliminary phase I vaccination trials in humans using NoV VLPs have confirmed that they are safe, and can effectively stimulate IgG and IgA responses also in man [17-19].

Besides antibody-mediated adaptive immunity, response to pathogenic viruses also relies on the elicitation of cellular adaptive immunity [20].

Peripheral blood mononuclear cells (PBMCs) consist mainly of monocytes, T-cells, B-cells, smaller amounts of natural killer (NK) cells and dendritic cells (DCs) of both myeloid and plasmacytoid origin. Dendritic cells are key determinants of viral disease outcome; they activate immune responses during viral infection and direct T-cells toward distinct T-helper type responses [21].

Multivariate and multiparametric analyses have been shown to predict the innate and early adaptive immune response induced by a vaccine molecule in human monocyte-derived dendritic cells (MDDCs) as well as PBMCs using an *ex vivo* experimental setting. *In vitro* stimulations performed on total PBMCs provide indication on the overall activation response induced by the antigen, comparable to data generated on the MDDC subset of cells [22]. This system biology approach involves high-throughput technologies such as global gene expression profiling, multiplex analysis of cytokines and chemokines, and multiparameter flow cytometry, combined with computational modeling [13,22-26].

In the present study, recombinant VLPs have been produced by cloning and expressing the ORF2 gene from a GII.4 norovirus strain detected in Italy in 2000 [27], using the recombinant baculovirus expression system. The produced VLPs were used to evaluate the immune response induced after *ex vivo* stimulation of human PBMCs. The results obtained show the ability of NoV VLPs to induce activation and maturation of circulating antigen presenting cells derived from five independent donors.

## Methods

### Production of Norovirus VLPs

Total RNA of a GII.4 norovirus strain (Hu/GII.4/00/IT; Grimsby-like) was extracted from stools of an infected patient, identified during a gastroenteritis outbreak that occurred in Italy in 2000 [27], using the QIAmp Viral RNA Extraction kit (QIAgen Hilden, Germany). The cDNA was obtained using primer (dT)20 and SuperScriptTM III reverse transcriptase (First-Strand SuperScriptTM III Synthesis System, Life Technologies, Carlsbad, CA), following the manufacturer’s instructions. The cDNA was used to amplify the entire ORF2, by PCR using primers FWORF2 (5′-cgc cgg atc cat gaa gat ggc gtc gaa tga-3′), flanked by *BamH*I restriction enzyme site (underlined) and including the methionine codon (in bold) and RWORF2 (5′-ctc gag taa tgc acg cct gcg ccc cgt tcc-3′), flanked by *Xho*I restriction enzyme site (underlined) and including the stop codon, indicated in bold. The 1700 bp DNA fragment obtained was ligated into the pFastBac™1 (Life Technologies) that was introduced into *E. coli* DH10Bac (Life Technologies), yielding the DNA clone BacHu/GII.4/00/IT. Nucleotide sequence was determined (Acc. No. KC462195). The bacmide was transfected into Sf9 insect cells to produce a high titer baculovirus. When a diffuse cytopathic effect was observed, infected Sf9 monolayers were harvested, and VLPs were purified by ultracentrifugation through a 30% (wt/vol) sucrose cushion, followed by a CsCl (1.362 g/cm^3^) density gradient [28]. Proper folding of the purified NoV VLPs was confirmed by electron microscopy (data not shown). Absence of residual contaminating baculovirus was confirmed by both electron microscopy and SDS-PAGE analysis.

### ELISA

Polystyrene microwell plates (NUNC, Rochester, NY) were coated with purified VLPs, using a concentration of 0.01 μg/well. The optimal concentration of capture antigen was established by chessboard titration using an anti-NoV positive mouse hyperimmune serum (not shown). A second plate was coated with a baculovirus infected cell extract expressing an unrelated protein, as negative control. After blocking by non fat milk, the serum samples were added (1:100 of human sera and 1:1000 for hyperimmune mouse serum). Binding of antibodies to the VLPs was detected using an anti-human GII.4 strain (kindly provided by L Svensson, Sweden) or anti-mouse alkaline phosphatase-labeled (AP) antibody. All assays were repeated three times. The cut-off value was determined as the mean OD value of negative samples plus two times the standard deviation [29].

### PBMC donor subjects

Five healthy volunteers (4 females and 1 male), with a mean age of 30 years (range 25– 47) were enrolled into the study. Peripheral blood was collected from each subject in 2011, under informed consent, and processed at the National Cancer Institute of Naples, as approved by the Institutional Review Board.

### PBMCs preparation

Fresh human PBMCs were isolated by Ficoll-Hypaque density gradient centrifugation, and plated in 6-well plates at a concentration of approximately 1 × 10^7^ cells/well in a maximum volume of 3 ml/well. Isolated PBMCs were incubated for 24 hours (short-term culture) or for 6 days (medium-term culture) in RPMI 1640 medium (Life Technologies, Carlsbad, CA).

### Cell culture medium

PBMCs culture medium consisted of RPMI 1640 medium (Life Technologies) supplemented with 2mM L-glutamine (Sigma), 10% fetal calf serum (Life Technologies), and 2% penicillin/streptomycin (5,000 IU, and 5 mg per ml, respectively. MP Biomedicals, Segrate, Italy). Recombinant interleukin-2 (rIL-2; R&D Systems, Minneapolis, MN) was added at a concentration of 75 U/ml for medium-term culture (6 days).

### PBMCs stimulation with VLPs

PBMCs were pulsed with purified NoV VLPs (10 μg/ml) for 24 hours or 6 days. In the latter case, VLPs where added to PBMCs at day 0 and 3. Residual endotoxin activity, due to lipopolysaccharide (LPS) possibly present in NoV VLP preparation, was blocked by pre-incubation with polymyxin B sulfate (SIGMA) at a concentration of 10 μg/ml. The absence of interference with activation due to polymyxin B sulfate was verified as previously described [13]. In parallel, cells were pulsed with 8 μg/ml of LPS, as positive control; PBS was added to unstimulated PBMCs. At the end of incubation, PBMCs were harvested, washed with PBS without calcium and magnesium, and stained for phenotypic analysis by flow cytometry. Cell supernatants were collected to quantify cytokine production, by ELISA.

### Flow cytometry

Short-term culture of PBMCs were incubated for 30 min at 4°C with human monoclonal antibodies specific for CD3, CD40, CD80, CD83, CD86, HLA-DR, CD123, CD11c and CD14 (BD Pharmingen, San Diego, CA), washed and analyzed with a FACScalibur flow cytometer (BD Pharmingen).

Mononuclear cells were gated by their specific forward (FSC) and side (SSC) scatters, excluding dead cells and debris. Among selected mononuclear cells, peripheral blood dendritic cells (PBDCs) were identified as cells positive for HLA-DR and negative for CD3 and CD14. Within HLA-DR^+^CD3^-^CD14^-^ PBDCs, myeloid dendritic cells (mDCs) were subsequently defined as CD11c-positive and CD123-negative cells, whereas plasmacytoid dendritic cells (pDCs) were defined as CD123-positive and CD11c-negative cells. Alternatively, within selected mononuclear cells monocytes were identified as HLA-DR^+^CD3^-^CD14^+^ cells.

Data analysis was carried out with the WinMDI2.8 Software. A paired t test was performed, all p-values were two-tailed and considered significant if less than 0.05.

### Cytokine analysis

At the time of cell harvesting, supernatants were collected and analyzed. Cytokine production was assessed using the Instant ELISA system (Bender Medsystems) for quantitative detection of human cytokines, according to the manufacturer’s instructions. Data acquisition was performed using a Sirio-S ELISA reader. A paired t test was performed, all p-values were two-tailed and considered significant if less than 0.05.

## Results

### GII.4 NoV VLPs induce a maturation phenotype in PBMCs

**Figure 1:**
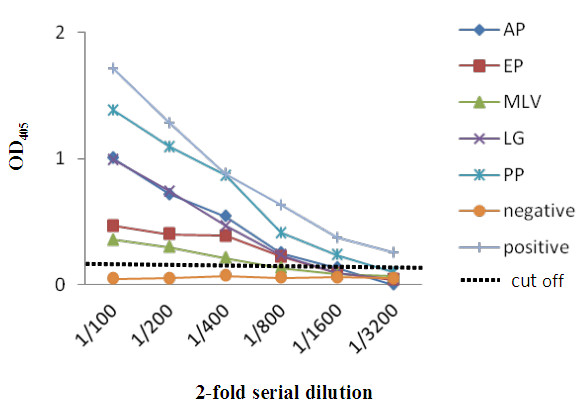
**Immunoreactivity of human GII.4 NoV VLPs with five human volunteers serum samples, human positive serum and negative serum using ELISA.** The cut-off value was determined as the mean OD value of negative samples plus two times the standard deviation [29].

Human sera were collected from five healthy volunteers, and were assayed against purified NoV VLPs, by ELISA (Figure 1). Sera were positive for antibodies against GII.4 NoV at dilutions between 1:100 to 1:3200, suggesting that the five subjects had experienced a previous infection with a GII.4 NoV strain or another norovirus genotype cross-reacting antigenically with GII.4.

In addition, Ficoll-Hypaque isolated PBMCs were prepared from the volunteers’ blood, and incubated with VLPs, or with LPS or PBS as positive and negative controls. After 24 hours stimulation, the expression of surface maturation/activation markers of immune cells, such as CD80, HLADR, CD83, CD86 and CD40 was evaluated by flow cytometry analysis.

**Figure 2:**
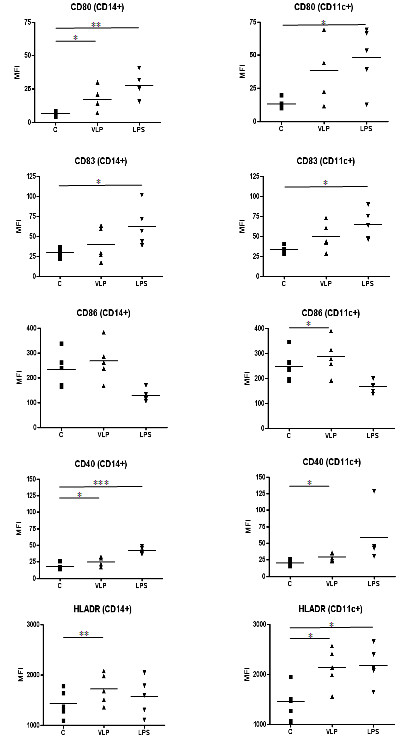
**Expression of surface maturation/activation markers, indicated as mean fluorescence intensity (MFI), induced by NoV VLPs and LPS on PBMCs from five healthy subjects.** * P < 0.05; ** P < 0.01; *** P < 0.001; c = control (un-stimulated PBMCs); CD14+ = CD14^+^ monocytes; CD11c + = CD11c^+^ mDCs.

VLP stimulation induced a trend of increased expression of all evaluated activation/maturation markers in circulating monocytes, as well as in myeloid dendritic cells (mDCs) (Figure 2).

In particular, an increased expression of CD80, CD40 and HLADR activation markers was induced in the CD14^+^ monocyte cell population (p < 0.05), whereas in CD11c^+^ mDC cells the CD40, CD86 and HLADR activation markers resulted significantly up-regulated (p < 0.05) (Figure 2). Only a weak activation signal was observed for CD123^+^ plasmacytoid dendritic cells (pDCs) (data not shown). No increase of CD86 was detected in either CD14^+^ monocytes or CD11c^+^ mDC cells upon stimulation with LPS, neither was HLADR induced in CD14+ cells, but the reasons for this remain unclear.

### Cytokine production by NoV VLP-loaded PBMCs

**Figure 3:**
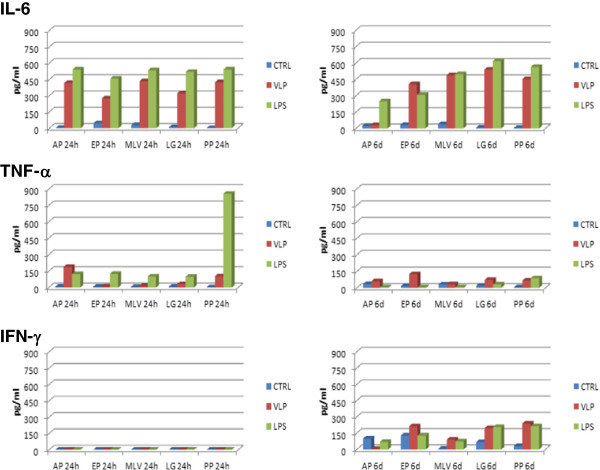
**Cytokine analysis in supernatants of PBMCs from five healthy subjects, induced by NoV VLPs and LPS.** Ranges of cytokine concentration detectable in the experimental conditions were: IL-6 (3–200 pg/ml), TNF-α (7–500 pg/ml), IFN-γ (1.6 - 100 pg/ml). Higher concentrations were deduced by analysis of diluted samples.

The level of interferon gamma (IFN-γ), tumor necrosis factor alpha (TNF-α), and IL-6 was determined in the supernatant of PBMCs loaded with NoV-VLPs *ex vivo*, after 24 hours or 6 days of incubation, except for IL-2 whose level was only determined at 24 hours. The results reported in Figure 3 show that NoV-VLPs induced significant production of IL-6 (p < 0.05). In particular, IL-6 level at 24 hours was significantly higher in VLP-stimulated PBMCs than in unstimulated PBMCs (p < 0.001), and the production of IL-6 persisted for the 6 days of induction (p < 0.05). No IFN-γ production was observed at 24 hours, and the cytokine level only increased after 6 days incubation for three of the five subjects (i.e. EP, LG, PP) (Figure 3). On the other hand, TNF-α induction was only detected for some of the donors at either 24 (i.e. AP, PP) or 6 days (i.e. EP, PP) (Figure 3), whereas no IL-2 production was induced in any subject by NoV-VLPs at 24 hours (data not shown). The overall results indicate that NoV-VLPs induced activation of PBMCs, which is coupled with the production of IL-6, IFN-γ and TNF-α.

## Discussion

The nature of immunity to norovirus is a key determinant if considering the prospect of future prevention of disease by possible vaccines. To date, little is known about the protective immune response elicited by norovirus infection, that is largely due to the lack of cell culture systems for noroviruses pathogenic to humans.

In the absence of *in vitro* replicating viruses, VLPs represent a useful tool for investigating virus-ligand interactions and the anti-viral immune response, since they strictly resemble infectious viruses both antigenically and morphologically [4,15,28]. VLPs might also be suitable for vaccination against NoV infection [19], similar to vaccines already in use for other viruses such as papillomavirus [30].

In the present study, we used a baculovirus expression system to generate VLPs from a NoV GII.4 strain identified during a major gastroenteritis outbreak in Italy in 2000 [27]. As previously reported by others [31-33], also the VLPs described in this study induced a strong immune response in Balb/c mice, eliciting elevated antibody levels (data not shown). The VLPs prepared in this study persisted during stressful CsCl centrifugation and proved antigenically stable at 4°C. They were thus suitable for investigating the cell-mediated immune response to GII.4 norovirus, which was done using peripheral blood cells from five asymptomatic adult volunteers. Although these subjects had not been affected by gastroenteritis in the 4 weeks before testing, they all resulted positive for NoV-antibodies in a NoV-VLP based ELISA test, indicating a previous infection with this or a similar norovirus genotype. This observation is not surprising since GII.4 is a pandemic genotype, and is in line with previous findings that between 90-100% healthy adult humans are seropositive for norovirus [3,4,34].

The innate and early adaptive immune response induced by GII.4 NoV-VLPs was evaluated on *ex vivo* stimulated PBMCs from the volunteers by means of activation/maturation phenotype analysis and cytokine production analysis. NoV-VLPs induced an increased expression of activation markers and co-stimulatory molecules in circulating APCs, particularly in CD14^+^ monocyte and CD11^+^ mDC cell populations. In particular, PBMC stimulation resulted in increased expression of surface activation/maturation markers (i.e. CD80, CD86, CD40 and HLA-DR), whose role in the initiation of the immune response is well defined. Both CD80 and CD86 are able to prime T cells, providing a co-stimulatory signal necessary for T cell activation and survival [35,36]. In addition, CD40 expression on antigen-presenting cells is known to be enhanced by CD40L on activated T cells, in a loop of induction of the immune response [37]. Finally, the HLA-DR molecule, which represents a ligand for the T-cell receptor (TCR), is up-regulated as well in response to antigenic stimuli [38]. The overall results provide interesting clue on the responsiveness of *ex vivo* loaded circulating APCs.

Moreover, PBMC stimulation resulted in increased production of specific cytokines, such as TNF-α, IL-6, whose level persisted for the 6 days of stimulation, and IFN-γ, whose level increased only after prolonged stimulation.

Several studies have been performed to identify the cytokine profile induced by NoV. In particular, an investigation on the T-cell response, induced in humans after a challenge with a GII.2 norovirus strain, identified a predominant Th1 CD4-dependent cell response, characterized by significant IFN-γ secretion [39]. Moreover, experimental infection of gnotobiotic piglets with a GII.4 human NoV strain was also reported to induce both antibodies and Th1/Th2 cytokine responses, locally and systemically [40]. These authors detected persistently higher Th1-specific cytokines (low transient IFN-γ and high IL-12) in infected pig sera, but also Th2-specific IL-4 and IL-6 cytokines, although to a lower level. Notably, a delayed IFN-α response was also evident [18].

Several authors suggest that response to norovirus infection mainly involves IFN-γ secretion by CD4 Th1 T cells [17,23,41,42].

Since the production of cytokines was not investigated in isolated cell subpopulations in the present study, no conclusion can be drawn about a specific pattern of Th1 versus Th2 response upon stimulation with GII.4 VLPs. However, our present observations suggest that despite an initial status characterized by IL-6 production, a prolonged stimulation may induce viable T cells to produce IFN-γ, implying that NoV-VLPs might activate autologous T cells.

The low reactivity shown by ELISA testing of some donors’ sera may be due to the antigenic differences between the strain(s) which had infected the five subjects in the past and the virus genotype corresponding to the VLPs used. The observation that IFN-γ became detectable only at later time points after *in vitro* stimulation might either indicate a switch in cytokine production or a low rate of secretion of this cytokine that would require a longer time to reach a detectable concentration in the culture supernatant.

## Conclusions

The present study on norovirus VLPs supports earlier findings, confirming that NoV VLP administration can specifically activate human PBMCs *ex vivo*.

Further studies are needed to clarify the reported lack of a long-lasting protective immune response following natural infection in man.

## Competing interests

The authors declare that they have no competing interests of either financial or non-financial nature regarding the work described in the present manuscript and its publication.

## Authors’ contributions

EP drafted the first version of the manuscript, designed experiments, analyzed and interpreted data, conducted serological testing, and participated to production of VLP and experiments for PBMC activation. AP analyzed PBMC data, participated to experiments for PBMC activation and contributed to a final draft. IDB participated to production of VLP and contributed to final draft preparation. LB and FMB designed experiments, and participated to final draft preparation. FMR supervised the activities, and reviewed the manuscript. All authors read and approved the final manuscript, and agreed with the conclusions of the work. None of the authors has any conflict of interest with the work being presented.

## Authors’ information

E Ponterio BSc, PhD, post-doc; I Di Bartolo BSc, PhD researcher; FM Ruggeri BSc, PhD Chief of Unit, Viral Zoonoses Unit, Department of Veterinary Public Health and Food Safety, Istituto Superiore di Sanità, Rome, Italy. L Buonaguro MD, Chief of laboratory; FM Buonaguro MD, Chief of Unit, and A Petrizzo BSc, PhD, post-doc, Laboratory of Molecular Biology and Viral Oncology, Department of Experimental Oncology, Istituto Nazionale per lo Studio e la Cura dei Tumori “Fondazione Pascale” - IRCCS, Naples, Italy.
